# Supplementary material for: The cost- effectiveness of early dental visit in infants and toddlers focused on regional deprivation in South Korea: A retrospective cohort study
Source: PLoS One. 2022 Jun 13;17(6):e0269770. doi: 10.1371/journal.pone.0269770 (PMC9191701; doi:10.1371/journal.pone.0269770)
Supplement: Attachment — Submitted filename: PONE-D-20-34952_R2-CG comments.pdf [file pone.0269770.s009.pdf]

PLOS ONE

The cost- effectiveness of early dental visit in infant and toddlers focused on regional deprivation in South Korea: A retrospective cohort study

--Manuscript Draft--

**Manuscript Number:**	PONE-D-20-34952R2
**Article Type:**	Research Article
**Full Title:**	The cost- effectiveness of early dental visit in infant and toddlers focused on regional deprivation in South Korea: A retrospective cohort study
**Short Title:**	The cost- effectiveness of early dental visit in infants and toddlers focused on regional deprivation in Korea
**Corresponding Author:**	Sun-Mi Kim, Ph.D<br>Wonkwang Health Science University<br>Iksan, KOREA, REPUBLIC OF
**Keywords:**	early childhood caries; economic evaluation; regional deprivation; Incremental cost-effectiveness ratio
**Abstract:**	Background : The aims of this study are to evaluate the cost-effectiveness of early dental visits (EDVs) and to investigate how regional deprivation impacts the economic evaluation.<br>Methods : This study used the South Korea National Health Insurance database, which included medical claim data and voluntary-based oral examination data. A retrospective cohort study was designed and measured all oral treatments, costs, and number of visits for 208,969 children who underwent oral examination for infants and toddlers from 2007 to 2014. The cost-effectiveness was measured using the incremental cost-effectiveness ratio, and the T-health index was used as the measurement for effectiveness. Regarding the distribution of dental hospitals/clinics, incentive based dental polices for either dental providers or patients are needed that will assure the delivery of dental care despite spatial inequality.<br>Results : The results of this study showed that EDVs were cost-effective and that children who participated in EDVs had better oral health (T-health-2 index difference 0.32 point in most deprived regions)and needed 5 USD less costly dental treatments than those who did not have EDVs. The cost-effectiveness of EDVs varied according to the level of regional deprivation and was the highest in the most deprived regions.<br>Conclusions : The study findings suggested that the provision of oral examination for infants and toddlers was a cost-effective dental policy. Additionally, EDVs were more effective in children who resided in the most deprived regions, a finding that will led to the development of policy intervention to improve dental care despite spatial inequality for disadvantaged population groups.
**Order of Authors:**	Eunsuk Ahn
	Sun-Mi Kim, Ph.D
**Opposed Reviewers:**	Amit Arora<br>Western Sydney University<br><br>The field of study is similar.<br><br>Medicine and health sciences, Health care, Socioeconomic aspects of health, Oral medicine, Oral health et al.
**Response to Reviewers:**	Thank you for taking a close look.<br>I made a mistake that should not be overlooked. As you said, we have corrected the title of the online system.<br><br>"The cost- effectiveness of early dental visit in infant and toddlers focused on regional deprivation in South Korea: A retrospective cohort study"
**Additional Information:**	
**Question**	**Response**

Powered by Editorial Manager® and ProduXion Manager® from Aries Systems Corporation

**Financial Disclosure**<br><br>Enter a financial disclosure statement that describes the sources of funding for the work included in this submission. Review the submission guidelines for detailed requirements. View published research articles from *PLOS ONE* for specific examples.<br><br>This statement is required for submission and **will appear in the published article** if the submission is accepted. Please make sure it is accurate.<br><br>**Unfunded studies**<br>Enter: *The author(s) received no specific funding for this work.*<br><br>**Funded studies**<br>Enter a statement with the following details:<br>• Initials of the authors who received each award<br>• Grant numbers awarded to each author<br>• The full name of each funder<br>• URL of each funder website<br>• Did the sponsors or funders play any role in the study design, data collection and analysis, decision to publish, or preparation of the manuscript?<br>• **NO** - Include this sentence at the end of your statement: *The funders had no role in study design, data collection and analysis, decision to publish, or preparation of the manuscript.*<br>• **YES** - Specify the role(s) played.<br><br>* typeset	The authors received no specific funding for this work.
**Competing Interests**<br><br>Use the instructions below to enter a competing interest statement for this submission. On behalf of all authors, disclose any competing interests that could be perceived to bias this work—acknowledging all financial support and any other relevant financial or non-financial competing interests.<br><br>This statement **will appear in the published article** if the submission is accepted. Please make sure it is	The authors have declared that no competing interests exist.

accurate. View published research articles from *PLOS ONE* for specific examples.<br><br>**NO authors have competing interests**<br><br>Enter: *The authors have declared that no competing interests exist.*<br><br>**Authors with competing interests**<br><br>Enter competing interest details beginning with this statement:<br><br>*I have read the journal's policy and the authors of this manuscript have the following competing interests: [insert competing interests here]*<br><br>* typeset	
**Ethics Statement**<br><br>Enter an ethics statement for this submission. This statement is required if the study involved:<br><br>• Human participants<br>• Human specimens or tissue<br>• Vertebrate animals or cephalopods<br>• Vertebrate embryos or tissues<br>• Field research<br><br>Write "N/A" if the submission does not require an ethics statement.<br><br>General guidance is provided below. Consult the submission guidelines for detailed instructions. **Make sure that all information entered here is included in the Methods section of the manuscript.**	Human Subject Research (involving human participants and/or tissue).<br>This study was approved by the Wonkwang University Institutional Review Board (WKIRB-201602-SB-007).

Powered by Editorial Manager® and ProduXion Manager® from Aries Systems Corporation

**Format for specific study types**<br><br>**Human Subject Research (involving human participants and/or tissue)**<br>• Give the name of the institutional review board or ethics committee that approved the study<br>• Include the approval number and/or a statement indicating approval of this research<br>• Indicate the form of consent obtained (written/oral) or the reason that consent was not obtained (e.g. the data were analyzed anonymously)<br><br>**Animal Research (involving vertebrate animals, embryos or tissues)**<br>• Provide the name of the Institutional Animal Care and Use Committee (IACUC) or other relevant ethics board that reviewed the study protocol, and indicate whether they approved this research or granted a formal waiver of ethical approval<br>• Include an approval number if one was obtained<br>• If the study involved *non-human primates*, add *additional details* about animal welfare and steps taken to ameliorate suffering<br>• If anesthesia, euthanasia, or any kind of animal sacrifice is part of the study, include briefly which substances and/or methods were applied<br><br>**Field Research**<br><br>Include the following details if this study involves the collection of plant, animal, or other materials from a natural setting:<br>• Field permit number<br>• Name of the institution or relevant body that granted permission	
**Data Availability**<br><br>Authors are required to make all data underlying the findings described fully available, without restriction, and from the time of publication. PLOS allows rare exceptions to address legal and ethical concerns. See the PLOS Data Policy and FAQ for detailed information.	Yes - all data are fully available without restriction

A Data Availability Statement describing where the data can be found is required at submission. Your answers to this question constitute the Data Availability Statement and **will be published in the article**, if accepted.<br><br>**Important:** Stating 'data available on request from the author' is not sufficient. If your data are only available upon request, select 'No' for the first question and explain your exceptional situation in the text box.<br><br>Do the authors confirm that all data underlying the findings described in their manuscript are fully available without restriction?	
**Describe where the data may be found in full sentences. If you are copying our sample text, replace any instances of XXX with the appropriate details.**<br><br>• If the data are **held or will be held in a public repository**, include URLs, accession numbers or DOIs. If this information will only be available after acceptance, indicate this by ticking the box below. For example: *All XXX files are available from the XXX database (accession number(s) XXX, XXX.)*.<br>• If the data are all contained **within the manuscript and/or Supporting Information files**, enter the following: *All relevant data are within the manuscript and its Supporting Information files.*<br>• If neither of these applies but you are able to provide **details of access elsewhere**, with or without limitations, please do so. For example:<br><br>*Data cannot be shared publicly because of [XXX]. Data are available from the XXX Institutional Data Access / Ethics Committee (contact via XXX) for researchers who meet the criteria for access to confidential data.*<br><br>*The data underlying the results presented in the study are available from (include the name of the third party*	Yes. doi: 10.6084/m9.figshare.14654442

Powered by Editorial Manager® and ProduXion Manager® from Aries Systems Corporation

and contact information or URL).<br>• This text is appropriate if the data are owned by a third party and authors do not have permission to share the data.<br><br>* typeset	
Additional data availability information:	Tick here if your circumstances are not covered by the questions above and you need the journal's help to make your data available.; Tick here if the URLs/accession numbers/DOIs will be available only after acceptance of the manuscript for publication so that we can ensure their inclusion before publication.

Submission of a paper to the PLOS ONE

Sun-Mi Kim Ph.D.
Wonkwang Health Science University
514, Iksan-daero, Iksan, Jeollabuk-do, Republic of Korea

May, 05, 2021

Dear PLOS One Editor(s),

We wish to submit a new manuscript titled, "The cost- effectiveness of early dental visit in infant and toddlers focused on regional deprivation in South Korea: A retrospective cohort study" for consideration by the PLOS ONE

In this paper, we report on the cost-effectiveness of early dental visits, which was defined as dental utilization for oral examination during infant period, using incremental cost effectiveness ratio. The authors seek to explore the cost-effectiveness of early dental visits (EDVs) and to investigate how regional deprivation impacts the economic evaluation. Through this study, we confirmed that early dental visits (EDVs) are an appropriate policy to efficiently use scarce medical resources from an individual as well as social point of view.

Thank you for your consideration of this manuscript.
Sincerely,

Sun-Mi,Kim

*Competing Interests : The authors have declared that no competing interests exist.

* Financial disclosure.: The authors received no specific funding for this work.

The control data is from South Korea, the Oral Examination for Infants and Toddlers (which is the official name of the policy and database, OEIT) and are owned by the Korea National

Organization (National Health Insurance. OEIT raw data can be accessed from the website https://nhiss.nhis.or.kr/bd/ab/bdaba010cv.do after requesting access. The authors did not have any special access to this data that others would not have.

Manuscript Click here to access/download;Manuscript;Manuscript.docx

The cost- effectiveness of early dental visit in infant and toddlers focused on regional deprivation in South Korea: A retrospective cohort study

Eunsuk, Ahn[1,#a], Sun-Mi, Kim[2*,#b]

[1] Department of Dental Hygiene, Daejeon Institute of Science and Technology, Daejeon, Republic of Korea

[2] Department of Dental Hygiene, Wonkwang Health Science University, North Jula, Republic of Korea

[#a] Current Address: Department of Dental Hygiene, Daejeon Institute of Science and Technology, 100 Hyechon-ro, Seo-gu, Daejeon, Republic of Korea

[#b] Current Address: Department of Dental Hygiene, Wonkwang Health Science University, 514 Iksan Dearo, Iksan, North Jula, Republic of Korea

[*] Corresponding Author: Sun-Mi, Kim

E-mail: dentalksm@naver.com

Abstract

Background : The aims of this study are to evaluate the cost-effectiveness of early dental visits (EDVs) and to investigate how regional deprivation impacts the economic evaluation.

Methods : This study used the South Korea National Health Insurance database, which included medical claim data and voluntary-based oral examination data. The subjects of this study included whole participants for oral examinations for infants and toddlers of the National Health Insurance Corporation. A retrospective cohort study was designed and measured all oral treatments, costs, and number of visits for 208,969 children (experimental group, 101,768; non-experimental group, 107,201) who underwent oral examination for infants and toddlers from 2007 to 2014. The cost-effectiveness was measured using the incremental cost-effectiveness ratio, and the T-health index was used as the measurement for effectiveness. In addition, the difference in the effect according to the level of regional deprivation was confirmed.

Results : The findings of this study showed that EDVs were cost-effective and that children who participated in EDVs had better oral health (T-health-2 index difference 0.32 point in most deprived regions) and needed 5 USD less costly dental treatments than those who did not have EDVs. The cost-effectiveness of EDVs varied according to the level of regional deprivation and was the highest in the most deprived regions.

Conclusions : The study findings suggested that the provision of oral examination for infants and toddlers was a cost-effective dental policy. Additionally, EDVs were more effective in children who resided in the most deprived regions, a finding that will led to the development of policy intervention to improve dental care despite spatial inequality for disadvantaged population groups. Regarding the distribution of dental hospitals/clinics, incentive based dental

polices for either dental providers or patients are needed that will assure the delivery of dental care despite spatial inequality.

Introduction

Early childhood caries (ECC) is defined as at least one caries lesion in a child younger than 5 years of age[1,2] and is recognized as a critical public health problem. Research findings show that the prevalence rate of ECC varies from population to population[2-5]. In the United States, the prevalence of dental caries among children 2-5 years of age was 27.9 % between 1999 and 2004[3]. It has been reported that 50% of Australian children under 12 years of age experience dental caries[4]. In Japan, the prevalence of dental caries was 25.9 % among children 3 years of age[2]. The prevalence rate of disadvantaged groups in developing countries was as high as 85 %[5]. In South Korea, the average prevalence rate of dental caries in 5-year-old children was 53.96 % in 2007-2014[6].

Previous studies reported that ECC not only affected oral health but also led to more pervasive consequences, such as costly treatment and issues with overall health, growth, and school performance[2,7,8]. ECC could negatively affect the quality of life for families[1,2,7] and could cause a significant economical and societal burden; however, it is preventable and potentially reversible in its early stages[9,10]. With timely and early intervention, it is possible to prevent and eliminate future dental caries and thus reduce dental-related costs.

The guidelines for the prevention of ECC include the following: self-care, use of professional services, and exposure to community interventions such as water fluoridation[4,9–11]. One of the prevention recommendations was the early dental visit (EDV), which was

reported to reduce both dental caries and the burden of dental-related costs[5,12], . The EDV was reported to be associated with fewer dental visits for children[5,11]. Other studies found a relationship between the EDV and dental costs or treatment use[13,14]. These studies emphasized that early prevention translated into a significant cost savings for dental care, especially for families at or below the poverty level. The research findings related to ECC led to the American Academy of Pediatric Dentistry, and the American Academy of Pediatrics recommendation[15] is that children have their first dental visit by their first birthday to prevent the incidence of ECC. In South Korea, the Oral Examination for Infants and Toddlers (which is the official name of the policy and database, OEIT) has been implemented as a part of the EDV since 2007. The OEIT is categorized into three age groups as follows: the 1st group is 18-24 months of age, the 2nd group is 42-48 months of age, and the 3rd group is 54-60 months of age.

Cost-effectiveness provides insight into the value of health interventions[16]. Findings of economic evaluations could help policy makers prioritize funding decisions. The significant benefits of the oral examination for infants and toddlers, no empirical study has been conducted to show the effectiveness of the oral examination for infants and toddlers in South Korea. Therefore, this study was conducted with the aim of confirming the effectiveness of the OEIT through the economic evaluation. In particular, it was attempted to determine the difference according to regional deprivation. And then, existing studies have used the dmft index, a conventional index for oral health, and have not confirmed oral health in terms of functionality, this study attempts to measure oral health using a new index that can measure functional oral health. The purpose of this study was twofold: (1) To investigate whether children aged 5 years and younger who do not receive EDV have worse oral health than those who do receive EDV; and (2) To find if the cost-effectiveness differs according to the level of community deprivation and to determine if the most deprived areas are associated with greater effectiveness .

Methods

Data and subjects

This study used the database of the National Health Insurance (NHI), which included medical claim data, enrollee information of NHI, and voluntary-based OEIT data. The OEIT data is a complete data for examination of infants and children in South Korea, and the study was conducted without applying a sampling technique. South Korea attained universal health insurance in 1989. Since that time, all citizens have been entitled to the comprehensive NHI. This means that the NHI dataset of this study represents the total South Korean population[17]. Enrollee data included general information of NHI enrollees such as demographics, premiums, medical fees, and location of residences. The OEIT dataset encompassed information related to the oral health examination, such as caries and periodontal status, and the response to a questionnaire, which included behavioral information such as usage of oral hygiene devices, dietary habits, and nutrition. Since the OEIT is a voluntary-based national recipient program, the oral examination data only contained 32.75 % of the total potential number of subjects.

Study design

This study was a retrospective cohort study. The study population consisted of 208,696 recipients in the 3rd group OEIT, who were 54-60 months of age at the time of the oral examination in 2011-2014. We traced retrospectively for a maximum of 60 months and collected relevant information from all events (dental visits) including types of dental treatments, service costs, and number of visits (Figure 1)[6]. We operationally defined the EDV

(exposed group) as the first dental visit in which dental services were utilized for oral examination prior to 24 months of age. In the non-EDV (non-exposed group), toddlers who did not undergo oral examination at 24 month were classified. A total of 208,969 were included as subjects. At this time, 101,768 people in the experimental group and 107,201 people in the non-experimental group were included, respectively. The study compared the cost-effectiveness between the 'exposed (EDV)' group and the 'non-exposed (non-EDV)' group.

This study was approved by the Wonkwang University Institutional Review Board (WKIRB-201602-SB-007).

Fig 1. Study population

Data analysis

Cost-effectiveness analysis

The economic evaluation compares two or more health intervention programs through the examination of costs of inputs and health outcomes[18]. There are 3 types of economic evaluations as follows: cost-effectiveness analysis (CEA), cost-benefit analysis, and cost-utility analysis[18]. A cost-effectiveness analysis compares the costs with natural oral health outcome units such as the T-Health index[19], the DMFT (or dmft) index [9], and the number of missing teeth[20]. For the Tissue Health (T-health) index, the higher the weight is given to the healthy tooth tissue, 4 points for healthy anterior teeth, 2 points for filling, 1 point for caries, and 0 points for loss[19]. A CEA is appropriate to inform decisions because it maintains health outcomes in its natural units rather than monetizing the outcomes[18,21,22]. Cost-effectiveness results can be expressed in costs per natural health units such as USD per the T-Health index or a cost-effectiveness ratio (CER)[16]. This study did not take into account the cost of loss of productivity and travel cost, so the cost-effectiveness of EDV and non-EDV compared by

calculating the direct medical cost covered by insurance for five years after the infant oral examination, excluding the indirect cost. The cost-effectiveness analysis of this study was conducted from a community level , and the results were stated using a CER. All analyses were performed using STATA ver. 11.0 (Stata Corp., College Station, TX, USA)

1) Costs

Costs in the cost-effectiveness analysis are generally classified into direct costs and indirect costs. Indirect costs encompass loss of productivity and/or quality of life. In this study, direct costs were only considered because the study population included only children, and thus a loss in productivity was not relevant. The study also did not include the indirect costs of parents or guardians that accompanied the children because the dataset did not provide the information of the guardians and the length of time of stay for dental services. Currently, the cost of dental services is limited to the range covered by health insurance including the cost of dental services, such as examinations and treatments, and travel costs, which include the expense of transportation from the residence to the dental clinic.

2) Effectiveness

We defined effectiveness via the T-health index per child, which was introduced by Bernabé et al[19]. The T-Health indicator is a weighted average of sound teeth, filled teeth, and teeth with some decay. The weights for T-Health represent the relative amounts of sound tissue in these three categories[23]. The T-Health index is based on the fact that a sound tooth contains more healthy dental tissue than a filled tooth and the latter can be considered to represent more healthy tissue than a decayed tooth because of the potential benefits of restorative treatment to the tooth shape and function[19,23]. In this study, we used 5 measures of the T-Health index of Bernabé et al[19]. In addition to the concept of the existing T-health

index, an index with a weight change as shown in figure 2 was used. This change in weight is because teeth with a smaller range of caries may have more healthy dental tissue than teeth with a wide range of filling. In this study, T-Health index is used to measure the magnitude of the effect. The T-Health index used in the study assigned a weight of 1 to a sound tooth, 0.10-0.50 to a filled tooth, 0.05-0.25 to a decayed tooth, and 0.00 to a missing tooth (Figure 2).

Fig 2. Five measures of the T-Health Index*

Note: *This study used the T-Health index of Bernabé et al[19]

3) Cost-effectiveness

The dmft index was defined as a decayed teeth have untreated legion, filled teeth have been repaired with restorative treatment, and missing teeth have been extracted caused by dental caries. It was included for comparison with T-Health index.

In this study, effectiveness was measured using the incremental cost-effectiveness ratio, which was defined as follows:

$$\mathrm{ICER} = \frac{cost_{EDV} - cost_{non-EDV}}{effect_{EDV} - effect_{non-EDV}}$$

where $cost_{EDV}$ and $cost_{non-EDV}$ per treated child represent the cost of the EDV group and the non-EDV group, respectively; $effect_{EDV}$ is the T-health index of the EDV group and $effect_{non-EDV}$ is the T-health index of the non-EDV group. The incremental cost-effectiveness ratio is a derivative concept and represents the incremental cost associated with additional unit of the T-health index.

Considered variables

We used gender, income, and regional variable for analysis. The income level was derived

from the health insurance premium of the National Health Insurance program, because NHI is mandated for all citizens, and the premium is imposed on the basis of income or assets of the insured. A variable considered to the regional variable was the composite deprivation index (CDI). The CDI was composed of five different subdomains (unemployment, poverty, housing, labor, and social relations) and was used to indicate the socioeconomic status of a municipality[24]. The CDI score was categorized into 3 groups using tertile as follows: low, middle, and high, with the low group being considered more affluent.

Results

Study population distribution according to socio-demographic characteristics

Table 1 shows the general characteristics of the subjects. The overall EDV rate was 48.70% (101,768) and was higher in the affluent group. As the income level increased, the EDV rate tended to increase. The differences in the rates of EDV between most poor and most affluent income levels were large in the regions with a high CDI score, which is most deprived areas. The number of dental visits was higher in the EDV group and increased as the CDI score decreased. In contrast, the cost of dental services was inversely related to the number of visits. The cost of dental services was higher in the non-EDV group than in the EDV group(Table 1).

Table 1. General characteristic of the subjects

Variables	Low CDI[*] (%)		Middle CDI (%)		High CDI (%)		Total
	Non-EDV[a]	EDV	Non-EDV	EDV	Non-EDV	EDV	(person)

Sex	Male	33.23	17.03	34.29	16.10	34.15	16.09	105,108
	Female	32.69	17.04	33.60	16.01	33.80	15.96	103,861
Income quintile	1st quartile (most poor)	9.71	4.53	11.81	4.96	13.22	5.37	34,517
	2nd quartile	11.24	5.69	13.88	6.29	15.47	7.02	41,454
	3rd quartile	8.00	4.30	9.10	4.51	9.54	4.66	27,923
	4th quartile	12.53	6.90	12.80	6.64	12.32	6.40	40,115
	5th quartile (most affluent)	24.45	12.64	20.31	9.71	17.40	8.60	64,960
Average no. of visits		4.85	5.23	4.86	5.30	4.79	5.09	
Cost of dental service[b]		226	221	234	232	246	235	

Asterisk (*) indicates that CDI: Composite deprivation index

[a] EDV-early dental visit

[b] unit-USD. Figures of sex and income levels are displayed as percentages.

The effectiveness of early dental visits

When comparing the T-Health index scores, the T-Health index scores of the EDV group were higher than those of its counterpart. The lowest scores occurred in the "middle CDI" regions, followed by the "low CDI" regions and the "high CDI" regions. Considering T-Health-2 as an example, the difference between the two groups (EDV vs. Non-EDV) in the “low” CDI regions was 0.25; the difference in the "middle" regions was 0.21; and the difference in the "high" regions was 0.32. However, the dmft index increased as the CDI score increased. The difference between the EDV group and the non-EDV group could be confirmed according to the type of T-health index whose measurement value was changed depending on whether or not dental treatment was received. However, the difference in the dmft index between the EDV and non-EDV groups was not consistent (Table 2).

Table 2. Effectiveness according to composite deprivation index

	Low CDI*		Middle CDI		High CDI	
	Non-EDV[a]	EDV	Non-EDV	EDV	Non-EDV	EDV
T-Health-10	17.29	17.52	17.31	17.50	17.27	17.56
T-Health-14	17.46	17.68	17.48	17.67	17.45	17.72
T-Health-6	17.12	17.36	17.13	17.34	17.09	17.39
T-Health-18	17.63	17.84	17.66	17.84	17.63	17.89
T-Health-2	16.95	17.20	16.96	17.17	16.91	17.23
dmft index	0.97	1.00	1.00	1.02	1.03	1.03

[a] EDV-early dental visit

Asterisk (*) indicates that CDI: Composite deprivation index

The cost-effectiveness of early dental visits

The ECC prevention effects of the EDV according to the regional deprivation index are shown in Figure 3. The incremental cost-effectiveness ratio of the T-health index using different weights showed cost-effectiveness for all weights.

At this time, negative ICER values that are shown in Figure 3 were due to positive effectiveness of natural units on teeth and the occurrence of treatment costs according to the condition of the teeth, which means a reduced cost between the non-EDV and EDV groups (Figure 3). The ICER varied according to the level of the CDI and was the highest in the high CDI regions. The ICER of the T-health index was distributed from 22.7 USD to 25.5 USD in the affluent regions. Among the middle CDI regions, the ICER of the T-health index showed from 1.6 USD to 10.9 USD, and in the regions with high CDI scores, the ICER was 34.5 USD to 42.7 USD. For the T-Health-10 index, the ICER of early dental visits in most deprived areas was approximately 39.1 USD, which indicated relatively high cost-effectiveness compared to 24.5 USD in the affluent areas.

Fig 3. ICER of early dental visits according to regional deprivation index

Discussion

Municipality socio-economic status could influence the oral health and accessibility of dental services, we also investigated how regional deprivation impacted incremental cost-effectiveness of EDVs. The results of this study showed that EDVs were cost-effective and that the effectiveness was the highest in the high CDI regions. In other words, these results mean that there is a cost-effectiveness in areas where there is a slope in the dental accessibility, and it suggests that the provision of such preventive services can contribute to oral health equity[5].

In this study, comparing the average number of dental visits per child between the non-EDV group and the EDV group, as shown in Table 1, it was found that the average number of dental visits in the EDV group was higher than that of the non-EDV group. However, when comparing the total cost of dental service, it was confirmed that the results were contradictory. The difference in total cost of dental service between the two groups tended to increase in areas where regional deprivation was severe, which was a result of supporting the previous researches[10,12].

The T-Health index was used instead of the dmft index to show the effectiveness of the ICER for EDVs. Many studies that involved economic evaluations used dmft/s, DMFT/s, or the potential cost savings associated with oral health outcomes[7,11,15,18,22]. However, the dmft/s or DMFT/s measures were less sensitive for detecting the progression of caries[25]. In addition, the functional oral health status was not well-reflected by the dmft index measures. Therefore, this study tried to improve these limitations by using the T-health index. As shown in Table 2, when the dmft index was used to evaluate the ICER, the effectiveness of the dmft index was higher in the EDV group, which is opposite to the expectation, because EDV users had more visits to the dental clinics for disease prevention. As a result, EDV users had several mild dental treatments, which resulted in a high score on the dmft. The dmft index does not

also determine a change (better or worse) in oral health due to treatment for oral disease. However, the T-Health index score reflects the changes in the quality of diseased teeth[26]. As observed in this study, a higher T-health index score indicates a healthier oral status, and using the T-Health index helped to conclude that better oral health was maintained through early dental visits.

In this study, we found that the EDV group incurred less dental costs than the non-EDV group. In spite of the fact that the number of dental visits for children who experienced EDVs was higher than for children who did not participate in EDVs, the average cost per dental visit showed that children who experienced EDVs spent less. It was thought as a result of the timely intervention of dental services, which reduce the overall costs associated with dental treatments, and that untreated dental diseases became more severe and costly because treatments were postponed[1,11,13−15,18]. The difference in dental costs between the two groups was the highest in the most-deprived regions. Previous studies reported that the behavior of individuals and available community resources were major determinants of oral health status[27-29]. It was assumed that children residing in deprived areas had inadequate access to dental services and, consequently, the oral health status of individuals was worse. When citing figures of children 5 years of age from Statistics Korea in 2010[30], the naïve cost savings of EDVs was estimated at 3.6 million USD. The cost savings of this study might be underestimated, because this study only included minimal direct costs.

The proportion of total oral health care expenditure among gross domestic products (GDP) was 0.5% in Korea in 2019, overtaking 0.3% in the UK, similar to those in Japan and France. [31], which created a great economic burden on society. The economic evaluation of oral health interventions would help justify the priority of oral health programs based on a coherent scientific method[22,25]. This study found that the cost-effectiveness differed according to the deprivation level of the community. The cost-

effectiveness of the ICER in the most deprived areas compared to affluent areas was approximately 1.6 times higher on average. The results of this study are even more meaningful when considering that the burden of oral diseases is not limited to economic burdens but includes pain, loss of productivity, and diminished quality of life. Since many researches are reported that children who come from a low socioeconomic status and disadvantaged community are at a relatively high risk for dental caries[28,29,32], providing timely oral services to children living in the most deprived communities result in greater cost savings and oral health improvements.

In conclusion, in terms of early intervention in the life course through early dental visits, providing oral examinations for infants and toddlers was a cost-effective policy. Additionally, EDVs were more effective in individuals who resided in the most deprived regions. This study is of the significance in that it suggested the necessity of a regional approach to the distribution of dental care by measuring the cost-effectiveness of EDV according to the level of regional deficiency using retrospective cohort data representing the Korean population. However, due to the limitation of the secondary data, there is a limitation in that indirect costs (travel cost, care cost etc), were not considered when calculating cost-effectiveness. Accordingly, in continued research, efforts to include appropriate variables that can sufficiently consider cost-effectiveness should be continued.

Supporting information

S1 File_dataset(ecc_sum).csv.(CSV)

S2 File_dataset(ecc_sem).csv. (CSV)

S3 File_dataset(ecc_min).csv. (CSV)

S4 File_dataset(ecc_mean).csv. (CSV)

S5 File_dataset(ecc_max).csv. (CSV)

Data Availability Statement

All relevant data are within the paper and its Supporting Information files.

References

1. Folayan MO, El Tantawi M, Schroth RJ, et al. Association between environmental health, ecosystem vitality, and early childhood caries. Front in Pediatrics. 2020;8:196. doi :10.3389/fped.2020.00196
2. Chen KJ, Duangthip D, Gao S, et al. Oral health policies to tackle the burden of early childhood caries: A Review of 14 Countries/Regions. Front. oral health. 2021;2:30. doi :10.3389/froh.2021.670154
3. Dye B a, Tan S, Smith V, et al. Trends in oral health status: United States, 1988-1994 and 1999-2004.; 2007.
4. Mejia GC, Ha DH. Dental caries trends in Australian school children. Aust Dent J. 2011;56(2):227-230. doi:10.1111/j.1834-7819.2011.01332.x.
5. Soares RC, da Rosa SV, Moysés ST et al. Methods for prevention of early childhood caries: Overview of systematic reviews. Int J Paediatr Dent. 2021;31(3):394-421. doi :10.1111/ipd.12766
6. Ahn E, Shin H. Effectiveness of oral examination for infants and toddlers: effects on subsequent utilization and costs. J Korean Acad Oral Heal. 2017;41(2):1-7.doi : 10.11149/jkaoh.2017.41.2.73
7. Nowak AJ, Casamassimo PS, Scott J, Moulton R. Do early dental visits reduce treatment and treatment cost for children? Pediatr Dent. 2014;36(7):489-493.
8. Baggio S, Abarca M, Bodenmann P, Gehri M, Madrid C. Early childhood caries in Switzerland: a marker of social inequalities. BMC Oral Health. 2015;15:82. doi:10.1186/s12903-015-0066-y.
9. Casamassimo PS, Thikkurissy S, Edelstein BL, Maiorini E. Beyond the dmft: the human and economic cost of early childhood caries. Journal of the American Dental Association (1939). 2009 Jun;140(6):650-657 doi:0.14219/jada.archive.2009.0250

10. Weintraub JA. Prevention of early childhood caries: a public health perspective. Community Dent Oral Epidemiol. 1998;26(1 Suppl):62-66.

11. Savage MF. Early preventive dental visits: effects on subsequent utilization and costs. Pediatrics. 2004;114(4):e418-e423. doi:10.1542/peds.2003-0469-F.

12. Tickle M, O'Neill C, Donaldson M et al. A randomised controlled trial to measure the effects and costs of a dental caries prevention regime for young children attending primary care dental services: the Northern Ireland Caries Prevention In Practice (NIC-PIP) trial. Health Technol Assess. 2016;20(71):1-96. doi: 10.3310/hta20710.

13. Sen B, Blackburn J, Morrisey M a, et al. Effectiveness of preventive dental visits in reducing nonpreventive dental visits and expenditures. Pediatrics. 2013;131(6):1107-1113. doi:10.1542/peds.2012-2586.

14. Beil H, Rozier RG, Preisser JS, Stearns SC, Lee JY. Effects of early dental office visits on dental caries experience. Am J Public Health. 2014;104(10):1979-1985. doi:10.2105/AJPH.2013.301325.

15. Ramos-Gomez FJ, Shepard DS. Cost-effectiveness model for prevention of early childhood caries. J Calif Dent Assoc. 1999;27(7):539-544. http://www.ncbi.nlm.nih.gov/pubmed/10530112. Accessed July 18, 2016.

16. Anopa Y, Macpherson L, McIntosh E. Systematic review of economic evaluations of primary caries prevention in 2- to 5-year-old preschool children. Value Health. 2020;23(8):1109-1118. doi: 10.1016/j.jval.2020.04.1823.

17. Aljunid S, Srithamrongsawat S, Chen W, et al. Health care data collecting, sharing, and using in Thiland, China, Mainland, South Korea, Taiwan, Japan, and Malaysis. Value Heal. 2012;15(1 Suppl):132-138.

18. Samnaliev M, Wijeratne R, Grace Kwon E, Ohiomoba H, Wai Ng M. Cost-effectiveness of a disease management program for early childhood caries. J Public

Health Dent. 2015;75(1):24-33. doi:10.1111/jphd.12067.

19. Bernabé E, Suominen-Taipale AL, Vehkalahti MM, Nordblad A, Sheiham A. The T-Health index: A composite indicator of dental health. Eur J Oral Sci. 2009;117(4):385-389. doi:10.1111/j.1600-0722.2009.00649.x.

20. Samnaliev M, Wijeratne R, Grace Kwon E, Ohiomoba H, Wai NM. Cost-effectiveness of a disease management program for early childhood caries. J Pub Health Dent. 2015;75(1):24–33. doi:10.1111/jphd.12067.

21. Manchanda K, Sampath N, Sarkar A. Evaluating the effectiveness of oral health education program among mothers with 6-18 months children in prevention of early childhood caries. Contemp Clin Dent. 2014;5(4):478. doi:10.4103/0976-237X.142815.

22. Koh R, Pukallus M, Kularatna S, et al. Relative cost-effectiveness of home visits and telephone contacts in preventing early childhood caries. 2015:560-568. doi:10.1111/cdoe.12181.

23. Marcenes WS, Sheiham A. Composite indicators of dental health: functioning teeth and the number of sound-equivalent teeth (T-Health). Community Dent Oral Epidemiol. 1993;21(6):374-378. doi:10.1111/j.1600-0528.1993.tb01102.x.

24. Shin H, Lee S, Chu J. Development of Composite Deprivation Index for Korea: The Correlation with Standardized Mortality Ratio. Prev Med Public Heal. 2009;42(6):392-402. doi:10.3961/jpmph.2009.42.6.392.

25. Tonmukayakul U, Sia K-L, Gold L, Hegde S, Silva AM de, Moodie M. Economic Models of Preventive Dentistry for Australian Children and Adolescents: A Systematic Review. Oral Health Prev Dent. 2015;13(6):481-495. doi:10.3290/j.ohpd.a35005.

26. Sheiham A, Maizels J, Maizels A. New composite indicators of Dental health. Community Dent Health. 1988;4(4):407-414. https://www.researchgate.net/publication/19675114_New_composite_indicators_of_D

ental_health. Accessed April 27, 2017.

27. Gennuso KP, Jovaag A, Catlin BB, Rodock M, Park H. Assessment of Factors Contributing to Health Outcomes in the Eight States of the Mississippi Delta Region. Prev Chronic Dis. 2016;13:150440. doi:10.5888/pcd13.150440.

28. Marmot M, Bell R. Social Determinants and Dental Health. Adv Dent Res. 2011;23(2):201-206. doi:10.1177/0022034511402079.

29. Prakash P, Subramaniam P, Durgesh BH, Konde S. Prevalence of early childhood caries and associated risk factors in preschool children of urban Bangalore, India: A cross-sectional study. Eur J Dent. 2012;6(2):141-152.

30. Korean statistical information service. Population and Housing Census. http://kosis.kr/statisticsList/statisticsList_01List.jsp?vwcd=MT_ZTITLE&parmTabId=M_01_01#SubCont. Published 2010. Accessed May 14, 2017.

31. Health at a Glance 2018 OECD Indicators, OECD Indicators - Google Play. https://www.oecd-ilibrary.org/docserver/4dd50c09-en.pdf?expires=1621676874&id=id&accname=guest&checksum=D88552B955E2D871BF55F10B5D0E9B4D. Accessed May 5, 2021.

32. Vargas CM, Ronzio C, Hayes K. Oral Health Status of Children and Adolescents by Rural Residence, United States. J Rural Heal. 2003;19(3):260-268. doi:10.1111/j.1748-0361.2003.tb00572.x.

Fig 1

Click here to access/download;Figure;fig 1.png

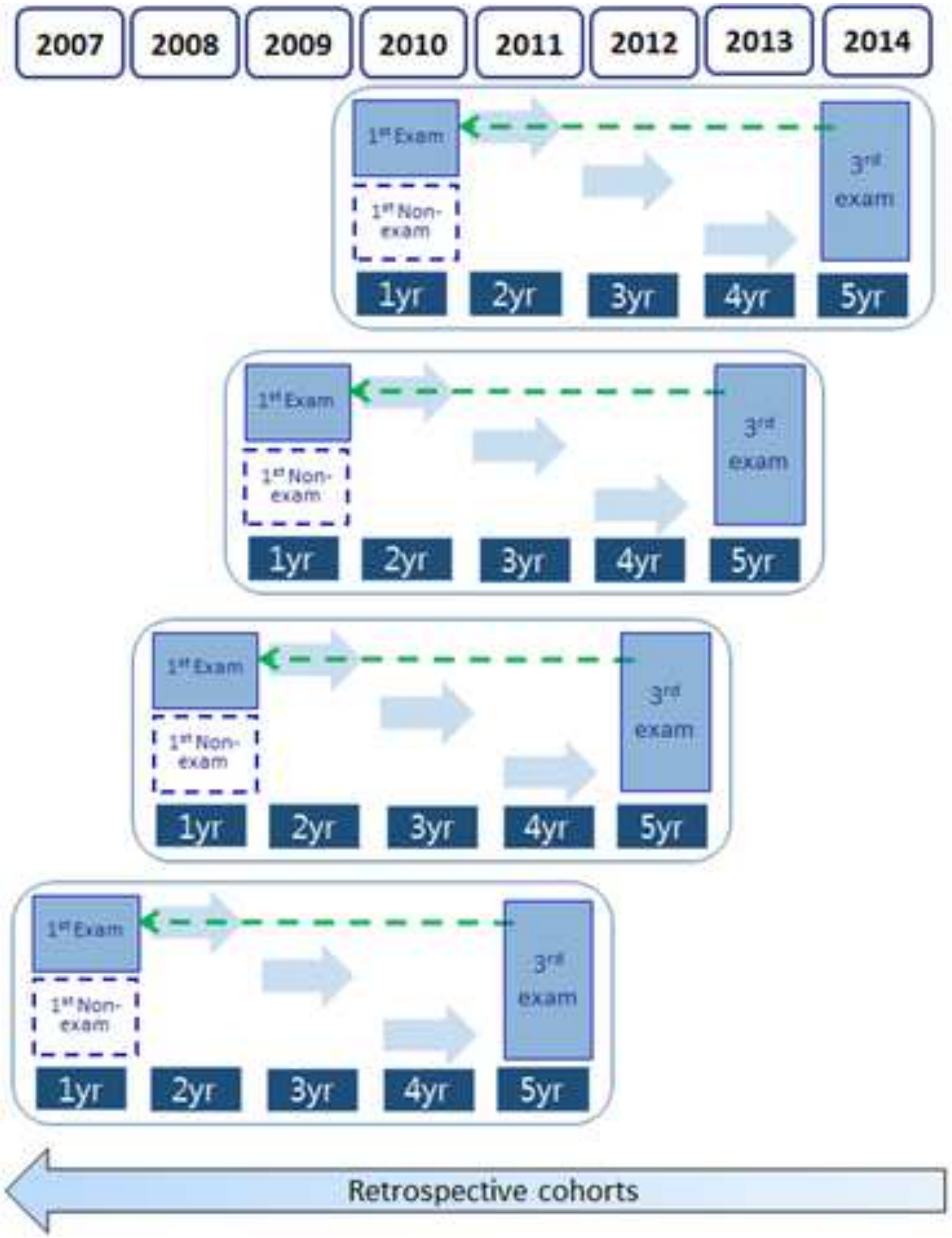

Fig 2

Click here to access/download;Figure;fig 2.tiff

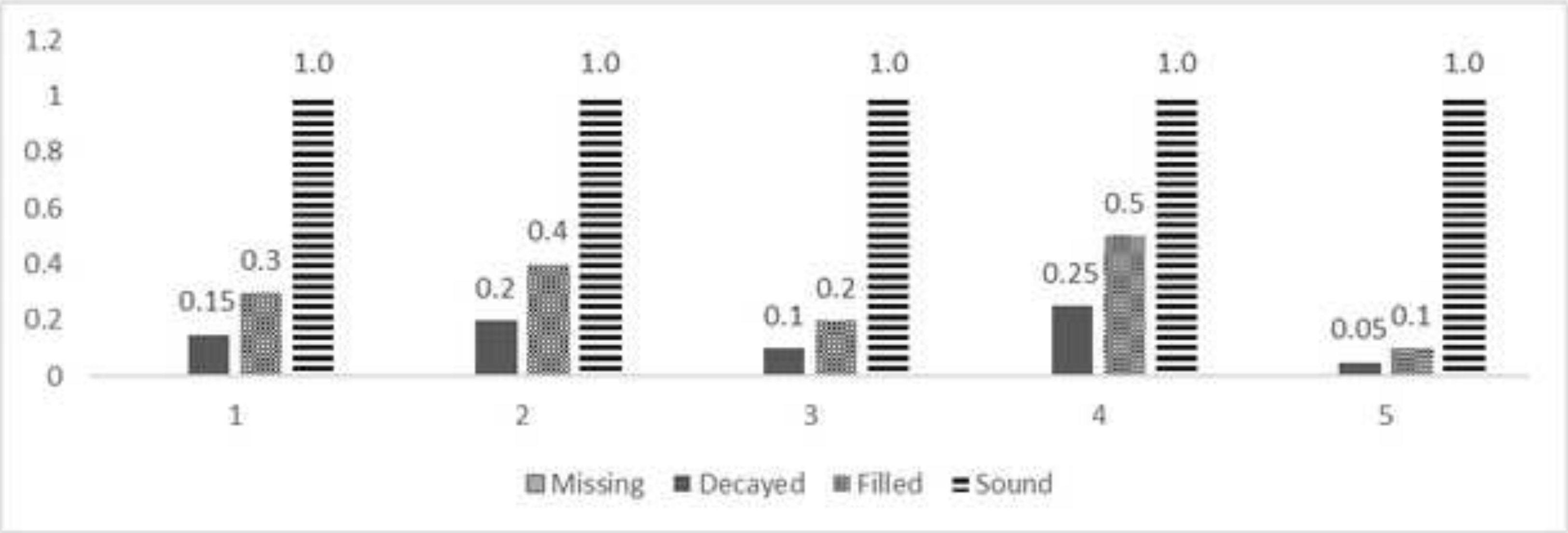

Fig 3

Click here to access/download;Figure;fig 3.tiff

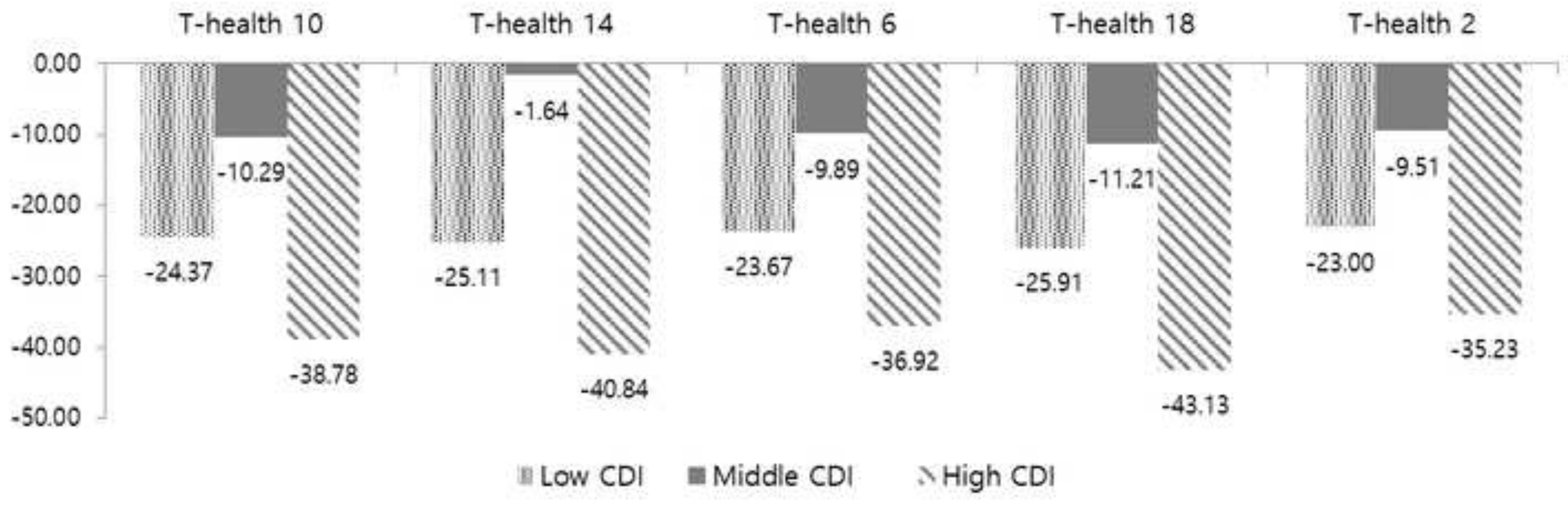

Click here to access/download
Supporting Information
S3_file_dataset(ecc_min).csv

Click here to access/download
Supporting Information
S2_file_dataset(ecc_sem).csv

S1_file_dataset(ecc_sum)

Click here to access/download
Supporting Information
S1_file_dataset(ecc_sum).csv

Click here to access/download
Supporting Information
S5_file_dataset(ecc_max).csv

S4_file_dataset(ecc_mean)

Click here to access/download

Supporting Information

S4_file_dataset(ecc_mean).csv

The cost- **effectiveness of early dental visit in infant and toddlers focused on regional deprivation in South Korea: A retrospective cohort study**

Eunsuk, Ahn[1,#a], Sun-Mi, Kim[2*,#b]

[1] Department of Dental Hygiene, Daejeon Institute of Science and Technology, Daejeon, Republic of Korea

[2] Department of Dental Hygiene, Wonkwang Health Science University, North Jula, Republic of Korea

[#a] Current Address: Department of Dental Hygiene, Daejeon Institute of Science and Technology, 100 Hyechon-ro, Seo-gu, Daejeon, Republic of Korea

[#b] Current Address: Department of Dental Hygiene, Wonkwang Health Science University, 514 Iksan Dearo, Iksan, North Jula, Republic of Korea

[*] Corresponding Author: Sun-Mi, Kim

E-mail: dentalksm@naver.com

Abstract

Background : The aims of this study are to evaluate the cost-effectiveness of early dental visits (EDVs) and to investigate how regional deprivation impacts the economic evaluation.

Methods : This study used the South Korea National Health Insurance database, which included medical claim data and voluntary-based oral examination data. The subjects of this study included whole participants for oral examinations for infants and toddlers of the National Health Insurance Corporation. A retrospective cohort study was designed and measured all oral treatments, costs, and number of visits for 208,969 children (experimental group, 101,768; non-experimental group, 107,201) who underwent oral examination for infants and toddlers from 2007 to 2014. The cost-effectiveness was measured using the incremental cost-effectiveness ratio, and the T-health index was used as the measurement for effectiveness. In addition, the difference in the effect according to the level of regional deprivation was confirmed.

Results : The findings of this study showed that EDVs were cost-effective and that children who participated in EDVs had better oral health (T-health-2 index difference 0.32 point in most deprived regions) and needed 5 USD less costly dental treatments than those who did not have EDVs. The cost-effectiveness of EDVs varied according to the level of regional deprivation and was the highest in the most deprived regions.

Conclusions : The study findings suggested that the provision of oral examination for infants and toddlers was a cost-effective dental policy. Additionally, EDVs were more effective in children who resided in the most deprived regions, a finding that will led to the development of policy intervention to improve dental care despite spatial inequality for disadvantaged population groups. Regarding the distribution of dental hospitals/clinics, incentive based dental

polices for either dental providers or patients are needed that will assure the delivery of dental care despite spatial inequality.

Introduction

Early childhood caries (ECC) is defined as at least one caries lesion in a child younger than 5 years of age[1,2] and is recognized as a critical public health problem. Research findings show that the prevalence rate of ECC varies from population to population[2-5]. In the United States, the prevalence of dental caries among children 2-5 years of age was 27.9 % between 1999 and 2004[3]. It has been reported that 50% of Australian children under 12 years of age experience dental caries[4]. In Japan, the prevalence of dental caries was 25.9 % among children 3 years of age[2]. The prevalence rate of disadvantaged groups in developing countries was as high as 85 %[5]. In South Korea, the average prevalence rate of dental caries in 5-year-old children was 53.96 % in 2007-2014[6].

Previous studies reported that ECC not only affected oral health but also led to more pervasive consequences, such as costly treatment and issues with overall health, growth, and school performance[2,7,8]. ECC could negatively affect the quality of life for families[1,2,7] and could cause a significant economical and societal burden; however, it is preventable and potentially reversible in its early stages[9,10]. With timely and early intervention, it is possible to prevent and eliminate future dental caries and thus reduce dental-related costs.

The guidelines for the prevention of ECC include the following: self-care, use of professional services, and exposure to community interventions such as water fluoridation[4,9–11]. One of the prevention recommendations was the early dental visit (EDV), which was

reported to reduce both dental caries and the burden of dental-related costs[5,12], . The EDV was reported to be associated with fewer dental visits for children[5,11]. Other studies found a relationship between the EDV and dental costs or treatment use[13,14]. These studies emphasized that early prevention translated into a significant cost savings for dental care, especially for families at or below the poverty level. The research findings related to ECC led to the American Academy of Pediatric Dentistry, and the American Academy of Pediatrics recommendation[15] is that children have their first dental visit by their first birthday to prevent the incidence of ECC. In South Korea, the Oral Examination for Infants and Toddlers (which is the official name of the policy and database, OEIT) has been implemented as a part of the EDV since 2007. The OEIT is categorized into three age groups as follows: the 1st group is 18-24 months of age, the 2nd group is 42-48 months of age, and the 3rd group is 54-60 months of age.

Cost-effectiveness provides insight into the value of health interventions[16]. Findings of ~~from~~ economic evaluations could help policy makers prioritize funding decisions. ~~Despite~~ T~~t~~he significant benefits of the oral examination for infants and toddlers, no empirical study has been conducted to show the effectiveness of the oral examination for infants and toddlers in South Korea. Therefore, this study was conducted with the aim of confirming the effectiveness of the OEIT through the economic evaluation. In particular, it was attempted to determine the difference according to regional deprivation. And then, existing studies have used the dmft index, a conventional index for oral health, and have not confirmed oral health in terms of functionality, this study attempts to measure oral health using a new index that can measure functional oral health. The purpose of this study was twofold: (1) To investigate whether children aged 5 years and younger who do not receive EDV have worse oral health than those who do receive EDV; and (2) To find if the cost-effectiveness differs according to the level of community deprivation and to determine if the most deprived areas are associated with greater

effectiveness .

Methods

Data and subjects

This study used the database of the National Health Insurance (NHI), which included medical claim data, enrollee information of NHI, and voluntary-based OEIT data. The OEIT data is a complete data for examination of infants and children in South Korea, and the study was conducted without applying a sampling technique. South Korea attained universal health insurance in 1989. Since that time, all citizens have been entitled to the comprehensive NHI. This means that the NHI dataset of this study represents the total South Korean population[17]. Enrollee data included general information of NHI enrollees such as demographics, premiums, medical fees, and location of residences. The OEIT dataset encompassed information related to the oral health examination, such as caries and periodontal status, and the response to a questionnaire, which included behavioral information such as usage of oral hygiene devices, dietary habits, and nutrition. Since the OEIT is a voluntary-based national recipient program, the oral examination data only contained 32.75 % of the total potential number of subjects.

Study design

This study was a retrospective cohort study. The study population consisted of 208,696 recipients in the 3rd group OEIT, who were 54-60 months of age at the time of the oral examination in 2011-2014. We traced retrospectively for a maximum of 60 months and

collected relevant information from all events (dental visits) including types of dental treatments, service costs, and number of visits (Figure 1)[6]. We operationally defined the EDV (exposed group) as the first dental visit in which dental services were utilized for oral examination prior to 24 months of age. In the non-EDV (non-exposed group), toddlers who did not undergo oral examination at 24 month were classified. A total of 208,969 were included as subjects. At this time, 101,768 people in the experimental group and 107,201 people in the non-experimental group were included, respectively. The study compared the cost-effectiveness between the 'exposed (EDV)' group and the 'non-exposed (non-EDV)' group.

This study was approved by the Wonkwang University Institutional Review Board (WKIRB-201602-SB-007).

Fig 1. Study population

Data analysis

Cost-effectiveness analysis

The economic evaluation compares two or more health intervention programs through the examination of costs of inputs and health outcomes[18]. There are 3 types of economic evaluations as follows: cost-effectiveness analysis (CEA), cost-benefit analysis, and cost-utility analysis[18]. A cost-effectiveness analysis compares the costs with natural oral health outcome units such as the T-Health index[19], the DMFT (or dmft) index [9], and the number of missing teeth[20]. For the Tissue Health (T-health) index, the higher the weight is given to the healthy tooth tissue, 4 points for healthy anterior teeth, 2 points for filling, 1 point for caries, and 0 points for loss[19]. A CEA is appropriate to inform decisions because it maintains health outcomes in its natural units rather than monetizing the outcomes[18,21,22]. Cost-effectiveness results can be expressed in costs per natural health units such as USD per the T-Health index

or a cost-effectiveness ratio (CER)[16]. This study did not take into account the cost of loss of productivity and travel cost, so the cost-effectiveness of EDV and non-EDV compared by calculating the direct medical cost covered by insurance for five years after the infant oral examination, excluding the indirect cost. The cost-effectiveness analysis of this study was conducted from a community level , and the results were stated using a CER. All analyses were performed using STATA ver. 11.0 (Stata Corp., College Station, TX, USA)

1) Costs

Costs in the cost-effectiveness analysis are generally classified into direct costs and indirect costs. Indirect costs encompass loss of productivity and/or quality of life. In this study, direct costs were only considered because the study population included only children, and thus a loss in productivity was not relevant. The study also did not include the indirect costs of parents or guardians that accompanied the children because the dataset did not provide the information of the guardians and the length of time of stay for dental services. Currently, the cost of dental services is limited to the range covered by health insurance including the cost of dental services, such as examinations and treatments, and travel costs, which include the expense of transportation from the residence to the dental clinic.

2) Effectiveness

We defined effectiveness via the T-health index per child, which was introduced by Bernabé et al[19]. The T-Health indicator is a weighted average of sound teeth, filled teeth, and teeth with some decay. The weights for T-Health represent the relative amounts of sound tissue in these three categories[23]. The T-Health index is based on the fact that a sound tooth contains more healthy dental tissue than a filled tooth and the latter can be considered to represent more healthy tissue than a decayed tooth because of the potential benefits of

restorative treatment to the tooth shape and function[19,23]. In this study, we used 5 measures of the T-Health index of Bernabé et al[19]. In addition to the concept of the existing T-health index, an index with a weight change as shown in figure 2 was used. This change in weight is because teeth with a smaller range of caries may have more healthy dental tissue than teeth with a wide range of filling. In this study, T-Health index is used to measure the magnitude of the effect. The T-Health index used in the study assigned a weight of 1 to a sound tooth, 0.10-0.50 to a filled tooth, 0.05-0.25 to a decayed tooth, and 0.00 to a missing tooth (Figure 2).

Fig 2. Five measures of the T-Health Index*

Note: *This study used the T-Health index of Bernabé et al[19]

3) Cost-effectiveness

The dmft index was defined as a decayed teeth have untreated legion, filled teeth have been repaired with restorative treatment, and missing teeth have been extracted caused by dental caries. It was included for comparison with T-Health index.

In this study, effectiveness was measured using the incremental cost-effectiveness ratio, which was defined as follows:

$$\mathrm{ICER} = \frac{cost_{EDV} - cost_{non-EDV}}{effect_{EDV} - effect_{non-EDV}}$$

where $cost_{EDV}$ and $cost_{non-EDV}$ per treated child represent the cost of the EDV group and the non-EDV group, respectively; $effect_{EDV}$ is the T-health index of the EDV group and $effect_{non-EDV}$ is the T-health index of the non-EDV group. The incremental cost-effectiveness ratio is a derivative concept and represents the incremental cost associated with additional unit of the T-health index.

Considered variables

We used gender, income, and regional variable for analysis. The income level was derived from the health insurance premium of the National Health Insurance program, because NHI is mandated for all citizens, and the premium is imposed on the basis of income or assets of the insured. A variable considered to the regional variable was the composite deprivation index (CDI). The CDI was composed of five different subdomains (unemployment, poverty, housing, labor, and social relations) and was used to indicate the socioeconomic status of a municipality[24]. The CDI score was categorized into 3 groups using tertile as follows: low, middle, and high, with the low group being considered more affluent. ~~The CDI score was categorized into 3 groups as follows: low, middle, and high, with the low group being considered more affluent.~~

Results

Study population distribution according to socio-demographic characteristics

Table 1 shows the general characteristics of the subjects. The overall EDV rate was 48.70% (101,768) and was higher in the affluent group. As the income level increased, the EDV rate tended to increase. The differences in the rates of EDV between most poor and most affluent income levels were large in the regions with a high CDI score, which is most deprived areas. The number of dental visits was higher in the EDV group and increased as the CDI score decreased. In contrast, the cost of dental services was inversely related to the number of visits. The cost of dental services was higher in the non-EDV group than in the EDV group(Table 1).

Table 1. General characteristic of the subjects

Variables		Low CDI* (%)		Middle CDI (%)		High CDI (%)		Total
		Non-EDV[a]	EDV	Non-EDV	EDV	Non-EDV	EDV	(person)
Sex	Male	33.23	17.03	34.29	16.10	34.15	16.09	105,108
	Female	32.69	17.04	33.60	16.01	33.80	15.96	103,861
Income quintile	1st quartile (most poor)	9.71	4.53	11.81	4.96	13.22	5.37	34,517
	2nd quartile	11.24	5.69	13.88	6.29	15.47	7.02	41,454
	3rd quartile	8.00	4.30	9.10	4.51	9.54	4.66	27,923
	4th quartile	12.53	6.90	12.80	6.64	12.32	6.40	40,115
	5th quartile (most affluent)	24.45	12.64	20.31	9.71	17.40	8.60	64,960
Average no. of visits		4.85	5.23	4.86	5.30	4.79	5.09	
Cost of dental service[b]		226	221	234	232	246	235	

Asterisk (*) indicates that CDI: Composite deprivation index

[a] EDV-early dental visit

[b] unit-USD. Figures of sex and income levels are displayed as percentages.

The effectiveness of early dental visits

When comparing the T-Health index scores, the T-Health index scores of the EDV group were higher than those of its counterpart. The lowest scores occurred in the "middle CDI" regions, followed by the "low CDI" regions and the "high CDI" regions. Considering T-Health-2 as an example, the difference between the two groups (EDV vs. Non-EDV) in the “low” CDI regions was 0.25; the difference in the "middle" regions was 0.21; and the difference in the "high" regions was 0.32. However, the dmft index increased as the CDI score increased. The difference between the EDV group and the non-EDV group could be confirmed according to the type of T-health index whose measurement value was changed depending on whether or not dental treatment was received. However, the difference in the dmft index between the EDV

and non-EDV groups was not consistent (Table 2).

Table 2. Effectiveness according to composite deprivation index

	Low CDI*		Middle CDI		High CDI	
	Non-EDV[a]	EDV	Non-EDV	EDV	Non-EDV	EDV
T-Health-10	17.29	17.52	17.31	17.50	17.27	17.56
T-Health-14	17.46	17.68	17.48	17.67	17.45	17.72
T-Health-6	17.12	17.36	17.13	17.34	17.09	17.39
T-Health-18	17.63	17.84	17.66	17.84	17.63	17.89
T-Health-2	16.95	17.20	16.96	17.17	16.91	17.23
dmft index	0.97	1.00	1.00	1.02	1.03	1.03

[a] EDV-early dental visit

Asterisk (*) indicates that CDI: Composite deprivation index

The cost-effectiveness of early dental visits

The ECC prevention effects of the EDV according to the regional deprivation index are shown in Figure 3. The incremental cost-effectiveness ratio of the T-health index using different weights showed cost-effectiveness for all weights.

At this time, negative ICER values that are shown in Figure 3 were due to positive effectiveness of natural units on teeth and the occurrence of treatment costs according to the condition of the teeth, which means a reduced cost between the non-EDV and EDV groups (Figure 3). ~~At this time, negative ICER values that are shown in Figure 3 were due to positive effectiveness and negative cost, which means a reduced cost between the non-EDV and EDV groups(Figure 3).~~

Formatted: Font color: Red

The ICER varied according to the level of the CDI and was the highest in the high CDI regions. The ICER of the T-health index was distributed from 22.7 USD to 25.5 USD in the

affluent regions. Among the middle CDI regions, the ICER of the T-health index showed from 1.6 USD to 10.9 USD, and in the regions with high CDI scores, the ICER was 34.5 USD to 42.7 USD. For the T-Health-10 index, the ICER of early dental visits in most deprived areas was approximately 39.1 USD, which indicated relatively high cost-effectiveness compared to 24.5 USD in the affluent areas.

Fig 3. ICER of early dental visits according to regional deprivation index

Discussion

Municipality socio-economic status could influence the oral health and accessibility of dental services, we also investigated how regional deprivation impacted incremental cost-effectiveness of EDVs. The results of this study showed that EDVs were cost-effective and that the effectiveness was the highest in the high CDI regions. In other words, these results mean that there is a cost-effectiveness in areas where there is a slope in the dental accessibility, and it suggests that the provision of such preventive services can contribute to oral health equity[5].

In this study, comparing the average number of dental visits per child between the non-EDV group and the EDV group, as shown in Table 1, it was found that the average number of dental visits in the EDV group was higher than that of the non-EDV group. However, when comparing the total cost of dental service, it was confirmed that the results were contradictory. The difference in total cost of dental service between the two groups tended to increase in areas where regional deprivation was severe, which was a result of supporting the previous researches[10,12].

The T-Health index was used instead of the dmft index to show the effectiveness of the ICER for EDVs. Many studies that involved economic evaluations used dmft/s, DMFT/s, or the potential cost savings associated with oral health outcomes[7,11,15,18,22]. However, the dmft/s or DMFT/s measures were less sensitive for detecting the progression of caries[25]. In addition, the functional oral health status was not well-reflected by the dmft index measures. Therefore, this study tried to improve these limitations by using the T-health index. ~~Since teeth with caries, missing teeth, and filled teeth have the same weight in the dmft index, it is difficult to evaluate the functional oral health status using this measure.~~ As shown in Table 2, when the dmft index was used to evaluate the ICER, the effectiveness of the dmft index was higher in the EDV group, which is opposite to the expectation, because EDV users had more visits to the

dental clinics for disease prevention. As a result, EDV users had several mild dental treatments, which resulted in a high score on the dmft. The dmft index does not also determine a change (better or worse) in oral health due to treatment for oral disease. However, the T-Health index score reflects the changes in the quality of diseased teeth[26]. As observed in this study, a higher T-health index score indicates a healthier oral status, and using the T-Health index helped to conclude that better oral health was maintained through early dental visits.

In this study, we found that the EDV group incurred less dental costs than the non-EDV group. In spite of the fact that the number of dental visits for children who experienced EDVs was higher than for children who did not participate in EDVs, the average cost per dental visit showed that children who experienced EDVs spent less. It was thought as a result of the timely intervention of dental services, which reduce the overall costs associated with dental treatments, and that untreated dental diseases became more severe and costly because treatments were postponed[1,11,13−15,18]. The difference in dental costs between the two groups was the highest in the most-deprived regions. Previous studies reported that the behavior of individuals and available community resources were major determinants of oral health status[27-29]. It was assumed that children residing in deprived areas had inadequate access to dental services and, consequently, the oral health status of individuals was worse. When citing figures of children 5 years of age from Statistics Korea in 2010[30], the naïve cost savings of EDVs was estimated at 3.6 million USD. The cost savings of this study might be underestimated, because this study only included minimal direct costs.

The proportion of total oral health care expenditure am

ong gross domestic products (GDP) was 0.5% in Korea in 2019, overtaking 0.3% in the UK, similar to those in Japan and France. [31], which created a great economic burden on society. The economic evaluation of oral health interventions would help justify the priority of oral health programs based on a coherent scientific method[22,25]. This study found that the

cost-effectiveness differed according to the deprivation level of the community. The cost-effectiveness of the ICER in the most deprived areas compared to affluent areas was approximately 1.6 times higher on average. The results of this study are even more meaningful when considering that the burden of oral diseases is not limited to economic burdens but includes pain, loss of productivity, and diminished quality of life. Since many researches are reported that children who come from a low socioeconomic status and disadvantaged community are at a relatively high risk for dental caries[28,29,32], providing timely oral services to children living in the most deprived communities result in greater cost savings and oral health improvements.

In conclusion, in terms of early intervention in the life course through early dental visits, providing oral examinations for infants and toddlers was a cost-effective policy. Additionally, EDVs were more effective in individuals who resided in the most deprived regions. This study is of the significance in that it suggested the necessity of a regional approach to the distribution of dental care by measuring the cost-effectiveness of EDV according to the level of regional deficiency using retrospective cohort data representing the Korean population. However, due to the limitation of the secondary data, there is a limitation in that indirect costs (travel cost, care cost etc), were not considered when calculating cost-effectiveness. Accordingly, in continued research, efforts to include appropriate variables that can sufficiently consider cost-effectiveness should be continued.

Supporting information

S1 File_dataset(ecc_sum).csv.(CSV)

S2 File_dataset(ecc_sem).csv. (CSV)

S3 File_dataset(ecc_min).csv. (CSV)

S4 File_dataset(ecc_mean).csv. (CSV)

S5 File_dataset(ecc_max).csv. (CSV)

Data Availability Statement

All relevant data are within the paper and its Supporting Information files.

References

1. Folayan MO, El Tantawi M, Schroth RJ, et al. Association between environmental health, ecosystem vitality, and early childhood caries. Front in Pediatrics~~. oral health~~. 2020;8:196. doi :10.3389/fped.2020.00196
2. Chen KJ, Duangthip D, Gao S, et al. Oral health policies to tackle the burden of early childhood caries: A Review of 14 Countries/Regions. Front. oral health. 2021;2:30. doi :10.3389/froh.2021.670154
3. Dye B a, Tan S, Smith V, et al. Trends in oral health status: United States, 1988-1994 and 1999-2004.; 2007.
4. Mejia GC, Ha DH. Dental caries trends in Australian school children. Aust Dent J. 2011;56(2):227-230. doi:10.1111/j.1834-7819.2011.01332.x.
5. Soares RC, da Rosa SV, Moysés ST et al. Methods for prevention of early childhood caries: Overview of systematic reviews. Int J Paediatr Dent. 2021;31(3):394-421. doi :10.1111/ipd.12766
6. Ahn E, Shin H. Effectiveness of oral examination for infants and toddlers: effects on subsequent utilization and costs. J Korean Acad Oral Heal. 2017;41(2):1-7.doi : 10.11149/jkaoh.2017.41.2.73
7. Nowak AJ, Casamassimo PS, Scott J, Moulton R. Do early dental visits reduce treatment and treatment cost for children? Pediatr Dent. 2014;36(7):489-493.
8. Baggio S, Abarca M, Bodenmann P, Gehri M, Madrid C. Early childhood caries in Switzerland: a marker of social inequalities. BMC Oral Health. 2015;15:82. doi:10.1186/s12903-015-0066-y.
9. Casamassimo PS, Thikkurissy S, Edelstein BL, Maiorini E. Beyond the dmft: the human and economic cost of early childhood caries. Journal of the American Dental Association (1939). 2009 Jun;140(6):650-657 doi:0.14219/jada.archive.2009.0250

10. Weintraub JA. Prevention of early childhood caries: a public health perspective. Community Dent Oral Epidemiol. 1998;26(1 Suppl):62-66.

11. Savage MF. Early preventive dental visits: effects on subsequent utilization and costs. Pediatrics. 2004;114(4):e418-e423. doi:10.1542/peds.2003-0469-F.

12. Tickle M, O'Neill C, Donaldson M et al. A randomised controlled trial to measure the effects and costs of a dental caries prevention regime for young children attending primary care dental services: the Northern Ireland Caries Prevention In Practice (NIC-PIP) trial. Health Technol Assess. 2016;20(71):1-96. doi: 10.3310/hta20710.

13. Sen B, Blackburn J, Morrisey M a, et al. Effectiveness of preventive dental visits in reducing nonpreventive dental visits and expenditures. Pediatrics. 2013;131(6):1107-1113. doi:10.1542/peds.2012-2586.

14. Beil H, Rozier RG, Preisser JS, Stearns SC, Lee JY. Effects of early dental office visits on dental caries experience. Am J Public Health. 2014;104(10):1979-1985. doi:10.2105/AJPH.2013.301325.

15. Ramos-Gomez FJ, Shepard DS. Cost-effectiveness model for prevention of early childhood caries. J Calif Dent Assoc. 1999;27(7):539-544. http://www.ncbi.nlm.nih.gov/pubmed/10530112. Accessed July 18, 2016.

16. Anopa Y, Macpherson L, McIntosh E. Systematic review of economic evaluations of primary caries prevention in 2- to 5-year-old preschool children. Value Health. 2020;23(8):1109-1118. doi: 10.1016/j.jval.2020.04.1823.

17. Aljunid S, Srithamrongsawat S, Chen W, et al. Health care data collecting, sharing, and using in Thiland, China, Mainland, South Korea, Taiwan, Japan, and Malaysia. Value Heal. 2012;15(1 Suppl):132-138.

18. Samnaliev M, Wijeratne R, Grace Kwon E, Ohiomoba H, Wai Ng M. Cost-effectiveness of a disease management program for early childhood caries. J Public

Health Dent. 2015;75(1):24-33. doi:10.1111/jphd.12067.

19. Bernabé E, Suominen-Taipale AL, Vehkalahti MM, Nordblad A, Sheiham A. The T-Health index: A composite indicator of dental health. Eur J Oral Sci. 2009;117(4):385-389. doi:10.1111/j.1600-0722.2009.00649.x.

20. Samnaliev M, Wijeratne R, Grace Kwon E, Ohiomoba H, Wai NM. Cost-effectiveness of a disease management program for early childhood caries. J Pub Health Dent. 2015;75(1):24–33. doi:10.1111/jphd.12067.

21. Manchanda K, Sampath N, Sarkar A. Evaluating the effectiveness of oral health education program among mothers with 6-18 months children in prevention of early childhood caries. Contemp Clin Dent. 2014;5(4):478. doi:10.4103/0976-237X.142815.

22. Koh R, Pukallus M, Kularatna S, et al. Relative cost-effectiveness of home visits and telephone contacts in preventing early childhood caries. 2015:560-568. doi:10.1111/cdoe.12181.

23. Marcenes WS, Sheiham A. Composite indicators of dental health: functioning teeth and the number of sound-equivalent teeth (T-Health). Community Dent Oral Epidemiol. 1993;21(6):374-378. doi:10.1111/j.1600-0528.1993.tb01102.x.

24. Shin H, Lee S, Chu J. Development of Composite Deprivation Index for Korea: The Correlation with Standardized Mortality Ratio. Prev Med Public Heal. 2009;42(6):392-402. doi:10.3961/jpmph.2009.42.6.392.

25. Tonmukayakul U, Sia K-L, Gold L, Hegde S, Silva AM de, Moodie M. Economic Models of Preventive Dentistry for Australian Children and Adolescents: A Systematic Review. Oral Health Prev Dent. 2015;13(6):481-495. doi:10.3290/j.ohpd.a35005.

26. Sheiham A, Maizels J, Maizels A. New composite indicators of Dental health. Community Dent Health. 1988;4(4):407-414. https://www.researchgate.net/publication/19675114_New_composite_indicators_of_D

ental_health. Accessed April 27, 2017.

27. Gennuso KP, Jovaag A, Catlin BB, Rodock M, Park H. Assessment of Factors Contributing to Health Outcomes in the Eight States of the Mississippi Delta Region. Prev Chronic Dis. 2016;13:150440. doi:10.5888/pcd13.150440.

28. Marmot M, Bell R. Social Determinants and Dental Health. Adv Dent Res. 2011;23(2):201-206. doi:10.1177/0022034511402079.

29. Prakash P, Subramaniam P, Durgesh BH, Konde S. Prevalence of early childhood caries and associated risk factors in preschool children of urban Bangalore, India: A cross-sectional study. Eur J Dent. 2012;6(2):141-152.

30. Korean statistical information service. Population and Housing Census. http://kosis.kr/statisticsList/statisticsList_01List.jsp?vwcd=MT_ZTITLE&parmTabId=M_01_01#SubCont. Published 2010. Accessed May 14, 2017.

31. Health at a Glance 2018 OECD Indicators, OECD Indicators - Google Play. https://www.oecd-ilibrary.org/docserver/4dd50c09-en.pdf?expires=1621676874&id=id&accname=guest&checksum=D88552B955E2D871BF55F10B5D0E9B4D. Accessed May 5, 2021.

32. Vargas CM, Ronzio C, Hayes K. Oral Health Status of Children and Adolescents by Rural Residence, United States. J Rural Heal. 2003;19(3):260-268. doi:10.1111/j.1748-0361.2003.tb00572.x.

Additional Editor Comments (if provided):

The authors appreciated your critical review comments. And we are very honored to give you the opportunity to submit your revised manuscript.

Line 52: US data reported is quiter old. Can it be updated?

- Yes, we have updated it to reflect your comments.

line 83: PLease the sentence construction. Seems like some missing information.

- We modified it to reflect your comments.

Lines 136 and 145 are repeated…. can be avoided

- Line 136 is the description of the variables used in the previous study, and line 145 is the definition of the operant variable in this study. We are very sorry that we did not make any special modifications to this.

Tsble 1 - check quintiles

- Yes, We checked it

DMFT index values have not been explained in the methods, so not clear why it is contradictory to expectations in Line 269.

- First of all, thanks for your comments. However, in the introduction and research method, the study using DMFT was explained earlier, and the discussion was to explain the difference between this study and other studies. However, as you commented, some parts of the sentences were not clearly expressed, so we have corrected them.

please read thoroughly to correct the grammar. Several sentences do not read well.

- We modified it to reflect your comments.

Comment: Anil Gumber

Paper is well written but lack methodological rigour. It is not clear to me in what perspective health economic evaluation is done. Author mentioned social perspective, but in paper it does not reflect in terms of loss of productivity of parents who care for their children in lieu of using dental services. The ICER is not presented well. I can't make out any thing from graphs representing negative ICER bars. The paper clearly lack focus in terms of analysis, costs and benefits. The authors need to present cost-effectiveness overall and then look by level of deprivation. The deprivation cut-off levels are arbitrary and to nullify it it should be use tertile or quartie or quintile break-up.

The authors appreciated your critical review comments. And we are very honored to give you the opportunity to submit your revised manuscript. In response to your comments, we have revised the manuscript as follows.

1. Author mentioned social perspective, but in paper it does not reflect in terms of loss of productivity of parents who care for their children in lieu of using dental services.

- The social point of view considered by the authors was the difference in the effect depending on the degree of deprivation in the region. Accordingly, the text has been modified as follows to convey a clearer meaning.

  Therefore, this study was conducted with the aim of confirming the effectiveness of the OEIT through the economic evaluation. In particular, it was attempted to determine the difference according to regional deprivation. …… …..; and (2) To find if the cost-effectiveness differs according to the level of community deprivation and to determine if the most deprived areas are associated with greater effectiveness .

- As you commented, it would have been better if the cost of lost productivity due to childc

are had been taken into account. Unfortunately, the authors were unable to fully account f or all variables in this study using secondary data. In this regard, we have added your co mments to the research methods and limitations of the research .

As previously stated, we will conduct further research in the future, taking into account y our comments. However, we ask for your understanding of some of the shortcomings of this study.

In consideration of your opinion, the text has been written as follows :

Methods

This study did not take into account the cost of loss of productivity and travel cost, so the cost-effectiveness of EDV and non-EDV compared by calculating the direct medical cost covered by insurance for five years after the infant oral examination, excluding the indire ct cost.

Discussion

However, due to the limitation of the secondary data, there is a limitation in that indirect costs (travel cost, care cost etc), were not considered when calculating cost-effectiveness. Accordingly, in continued research, efforts to include appropriate variables that can suffi ciently consider cost-effectiveness should be continued.

2. The ICER is not presented well. I can't make out any thing from graphs representing nega tive ICER bars.

- Thank you for your critical comments. We have made the following changes to reflect your comments.

  At this time, negative ICER values that are shown in Figure 3 were due to positive effectiveness of natural units on teeth and the occurrence of treatment costs according to the condition of the teeth, which means a reduced cost between the non-EDV and EDV groups (Figure 3).

3. The deprivation cut-off levels are arbitrary and to nullify it it should be use tertile or quartie or quintile break-up.

- Thank you for your critical comments following a detailed review. Even though our authors have already classified using the tertile, it seems that they did not describe the content sufficiently. We have made the following changes to reflect your comments.

  The CDI score was categorized into 3 groups using tertile as follows: low, middle, and hi gh, with the low group being considered more affluent.
