# Supplementary material for: The cost- effectiveness of early dental visit in infants and toddlers focused on regional deprivation in South Korea: A retrospective cohort study
Source: PLoS One. 2022 Jun 13;17(6):e0269770. doi: 10.1371/journal.pone.0269770 (PMC9191701; doi:10.1371/journal.pone.0269770)
Supplement: Attachment — Submitted filename: author_reponse_0523.docx [file pone.0269770.s007.docx]

Author’ Response

Thank you for a careful review. And we are very honored to give you the opportunity to submit your revised manuscript. Authors revised the manuscript to reflect the opinions of reviewers. Detailed revisions reflecting the opinions given by reviewers are indicated in the attached 'manuscript' file.

1. Title

- The title has been changed to reflect the reviewer's very good opinion. In more detail, the title has been changed to include the content of' the difference in the effect of oral examinations according to regional deficiency', which was the main interest of this study.

2. Abstracts

- The authors have divided the sections into background, methods, results, and conclusions. In addition, the research method and results were described more clearly in accordance with the characteristic of the academic paper, and a review was prepared by reflecting the purpose and results of the research.

3. Introduction

- The references mentioned in this study tend to be somewhat outdated, so the authors have updated them with the latest studies. In addition, the authors mentioned the differentiation of the T-health index used in the cost-effectiveness analysis.

4. Methods

- The authors have revised and described the characteristics and sizes of the samples used in this study to more clearly explain. However, the Oral Examination for Infants and Toddlers data used in this study is not a sample, but a population data in Korea. This is because sampling techniques were not required, so I mentioned that part in the text. The experimental group and the control group used in this study were described by adding clear explanations. Also, figure 1 has been added for better understanding in study design section.

- The description of the variables used in the cost-effectiveness analysis has been added as follows, 1) Each section was divided for the variables calculated in this study, such as cost, effectiveness, and cost-effectiveness; 2) Added explanations and references for 5 T-health indexes; 3) The unit of measurement was more clearly expressed.

5. Results

- In the case of Table 1, the% calculation standard has been revised and newly presented for better understanding. In addition, 5 T-health index descriptions have been added in Table 2.

6. Discussions

- In order to reveal the implications of the study, the authors have overall revised the discussion so that the results of this study can be compared with the existing studies. In the discussion section, the number of visits to the dentist and the total cost of dental care have been added. In addition, the technical section on the limitations and strengths of the research has been added.
