# Supplementary material for: The cost- effectiveness of early dental visit in infants and toddlers focused on regional deprivation in South Korea: A retrospective cohort study
Source: PLoS One. 2022 Jun 13;17(6):e0269770. doi: 10.1371/journal.pone.0269770 (PMC9191701; doi:10.1371/journal.pone.0269770)
Supplement: Attachment — Submitted filename: response to additional editor comments_1224.docx [file pone.0269770.s008.docx]

Additional Editor Comments (if provided):

The authors appreciated your critical review comments. And we are very honored to give you the opportunity to submit your revised manuscript.

Line 52: US data reported is quiter old. Can it be updated?

- Yes, we have updated it to reflect your comments.

line 83: PLease the sentence construction. Seems like some missing information.

- We modified it to reflect your comments.

Lines 136 and 145 are repeated…. can be avoided

- Line 136 is the description of the variables used in the previous study, and line 145 is the definition of the operant variable in this study. We are very sorry that we did not make any special modifications to this.

Tsble 1 - check quintiles

- Yes, We checked it

DMFT index values have not been explained in the methods, so not clear why it is contradictory to expectations in Line 269.

- First of all, thanks for your comments. However, in the introduction and research method, the study using DMFT was explained earlier, and the discussion was to explain the difference between this study and other studies. However, as you commented, some parts of the sentences were not clearly expressed, so we have corrected them.

please read thoroughly to correct the grammar. Several sentences do not read well.

- We modified it to reflect your comments.
