# Supplementary material for: The cost- effectiveness of early dental visit in infants and toddlers focused on regional deprivation in South Korea: A retrospective cohort study
Source: PLoS One. 2022 Jun 13;17(6):e0269770. doi: 10.1371/journal.pone.0269770 (PMC9191701; doi:10.1371/journal.pone.0269770)
Supplement: Attachment — Submitted filename: Response to Reviewers_220512.docx [file pone.0269770.s010.docx]

Response to Reviewers

We thank the reviewers for their thorough review.

We have reviewed and revised the entire sentence as well as the part of the review comments.

The authors would like to thank the editorial staff once again for their efforts.

In addition, first author’s affiliation has been changed and the corresponding information is added.

Ahn Eunsuk

Division of Climate Change and Health Protection. Korea Disease Control and Prevention Agency.
