# Supplementary material for: The cost- effectiveness of early dental visit in infants and toddlers focused on regional deprivation in South Korea: A retrospective cohort study
Source: PLoS One. 2022 Jun 13;17(6):e0269770. doi: 10.1371/journal.pone.0269770 (PMC9191701; doi:10.1371/journal.pone.0269770)
Supplement: Attachment — Submitted filename: Reviewer comment.docx [file pone.0269770.s006.docx]

**Comment: Kasahun Girma Tareke**

The authors conducted a research entitled “The cost-effectiveness of oral examination for infants and toddlers”. The research is a very interesting work. However, there is a need to make amendment of important parts before accepted for publication. Here find the suggested comments to improve the paper.

**Abstract**

1. Please make partition for background, methods, results and conclusions.
   1.1. Make your methods clear (i.e., sample size for each cases and controls, how you calculated the sample size, sampling technique, methods of data extraction from the data base, data analysis, etc.)
   1.2. Make your results clear based on your research objective (s)/question (s). The result section seems like conclusions. Put the numeric/figurative findings for each of the findings.
   1.3. Make your conclusions consistent with results. This might be corrected or becomes clear once you make correction of presentation of research findings.

**Introduction**

1. Good! However, make your research aims consistent with your title; given that the title only focused on cost-effectiveness but not included the first purpose of your study.

**Materials and method**

1. Please switch to “Methods”.
2. Please give descriptions of your study area/setting, and also the description of the project being implemented since 2007. You have mentioned that “The OEIT is categorized into three age groups 75 as follows: the 1st group is 18-24 months of age, the 2nd group is 42-48 months of age, and the 76 3rd group is 54-60 months of age.” Please specify the type of interventions done for each group.
3. Study design: It is good that you mentioned the study design. However, define the cases and controls clearly.
4. Study populations: It is not clear the number of population among cases and controls. Therefore, please specify it.
5. Sampling and sample size calculation: Your study lacks any information about the samples, calculation formulas, sampling techniques, procedures, etc.
6. Eligibility criteria??? Think of secondary data source.
7. Data analysis: It is not clear about the software you were used to clean, enter or analyzed the data. Other, the type of cost-analysis you have done is not consistent with your title. Your title only focused on one of the cost-analysis; cost-effective analysis.
8. Please make a measurement for your variables (cost-effectiveness, cost-benefit, and cost utility, cost, effectiveness, etc.)
9. **Result:** Good but you had not presented findings for the research purpose 1 presented on introduction section. Clearly show it. Also not presented according to cost-effectiveness, cost-benefit and cost-utility.
10. **Discussion:** Please delete the first two sentences of discussion and incorporate it somewhere in the introduction section. Write your pertinent findings as a first paragraph of discussion, and discuss them each by each in the consecutive paragraphs. The third paragraph seems like conclusion. What was your base to say cost-effective at this rudimentary stage? Make your study findings and discussion consistent. On line 271 of your paper, you have discussed that the cost-effectiveness of the ICER in the most deprived areas compared to affluent areas was approximately 1.6 times higher on 272 averages. But, you had not put the odds ratio for your findings. Therefore, please amend your result section incorporating the odds ratio, P-value and confidence intervals.
11. **Please add strength and limitations of the study.** I think it might have limitations since you had used a secondary data or retrospective cohort study.
12. **Conclusions:** Make a label “conclusions”. Make consistent with your objectives, pertinent findings and discussions.
13. **Please define the abbreviations**
14. **Declerations????**
15. **References:** You had used too old references. Please try to discuss your findings with the updated research findings.
